# Supplementary material for: Unified Brain MR-Ultrasound Synthesis using Multi-Modal Hierarchical Representations
Source: Med Image Comput Comput Assist Interv. Author manuscript; Available in PMC 2024 Apr 23. (PMC7615858; doi:10.1007/978-3-031-43999-5_43)
Supplement: Appendix [file EMS195482-supplement-Appendix.pdf]

# 6 Appendix

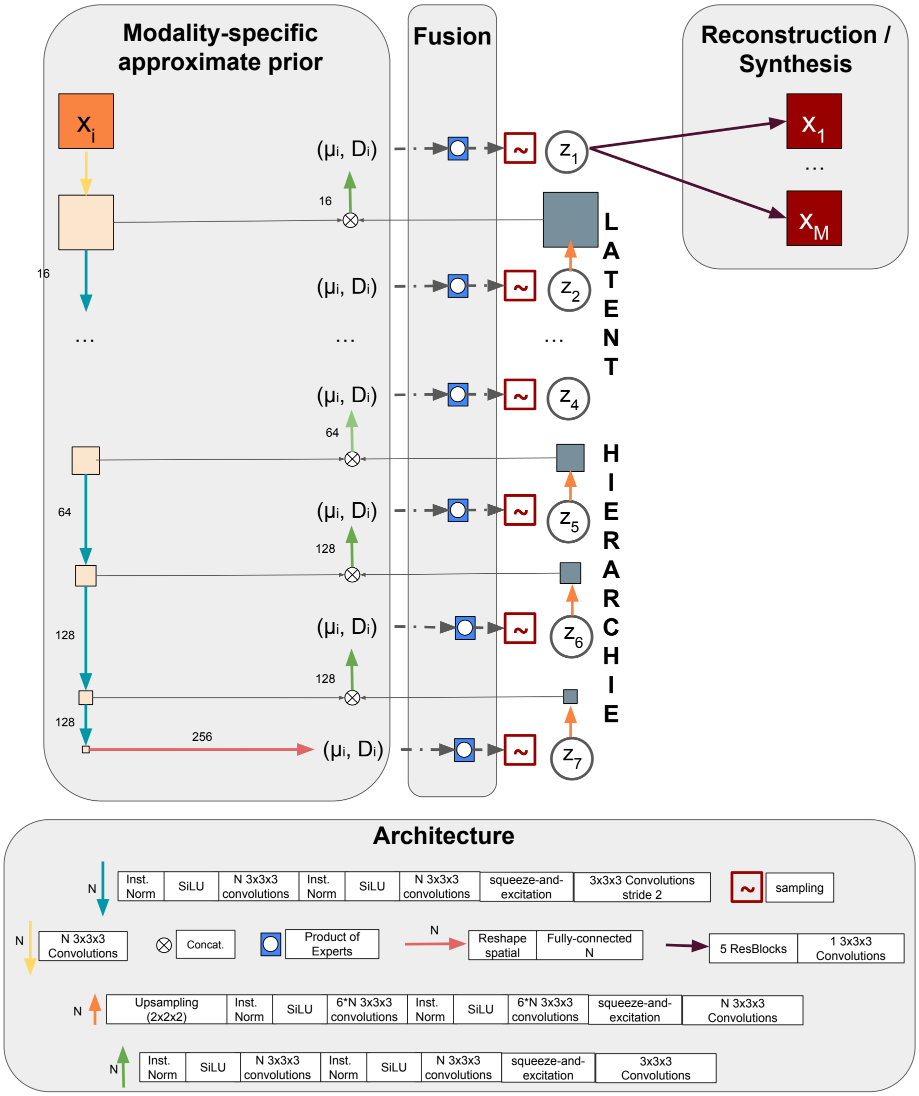


**Fig. 3.** Our multi-modal hierarchical variational auto-encoder (MHVAE). Only one modality encoder is shown. Products of experts are defined in the core manuscript.

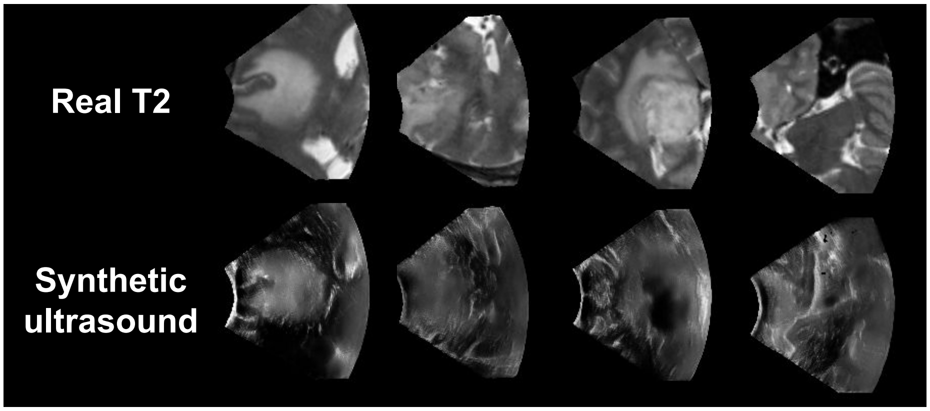


**Fig. 4.** Example of synthetic ultrasound images generated from T2 scans of the BraTS dataset.
